# How genomics and multi-modal AI are reshaping precision medicine

**DOI:** 10.3389/fmed.2025.1660889

**Published:** 2025-08-26

**Authors:** Han Zhuang

**Affiliations:** Ningbo Institute of Digital Twin, Ningbo, China

**Keywords:** precision medicine, multi-modal AI, disease, genomics, diagnostics

Precision medicine helps healthcare professionals design medical treatment for individuals for decades. And biomarker matching therapies has shown effective for survival rate improvement (1). In a study of metastatic tumors, 15.6% of CGP-tested patients received targeted therapy and CGP-guided treatment was correlated with prolonged progression-free survival [pooled hazard ratio (HR) = 0.63] (2). Today, we are facing an important turning point where the convergence of genomics and multi-modal artificial intelligence is transforming precision medicine into new ways to diagnose, treat, and prevent disease.

## Genomics as the cornerstone of precision medicine

Genomics are the foundations of precision medicine. The completion of the Human Genome Project in 2003 marked the beginning of a new era in healthcare, revealing how genetic variations influence disease diagnose, drug metabolism, and treatment response.

The impact of genomics on clinics is profound. Pharmacogenomic testing can guide dosing decisions for critical medications, preventing adverse reactions and treatment failures (3, 4). In oncology, tumor genomic profiling has revolutionized cancer care, enabling targeted therapies that specifically attack cancer cells based on their genetics (5). Hereditary cancer syndromes, and other genetic diseases can now be diagnosed by genetic techniques, allowing for early intervention and family screening (6). In neurology, researchers developed technique to detect biomarker of demyelinating disease (7). And for an example in infectious disease, researchers developed VirCapSeq-VERT, a positive selection system based on genetics for detecting RNA and DNA viruses (8).

However, genomics also showed the limitations of genetic determinism. The complex interactions between genes, environment, and human body means that genetic information alone cannot fully predict health conditions or optimal treatments. This realization has driven the start toward multi-modal approaches that consider genomics as the cornerstone while recognizing the need for other layers of biological and clinical information.

## Not only the genome: the multi-modal imperative

While genomics is foundation for precision medicine, we believe that genes alone can tell only part of the story. The human body is an intricate system where genetic intersects with environmental factors, lifestyle choices, and organs. Multi-modal AI can be one of our most powerful techniques to explore this complexity.

**Figure 1:**
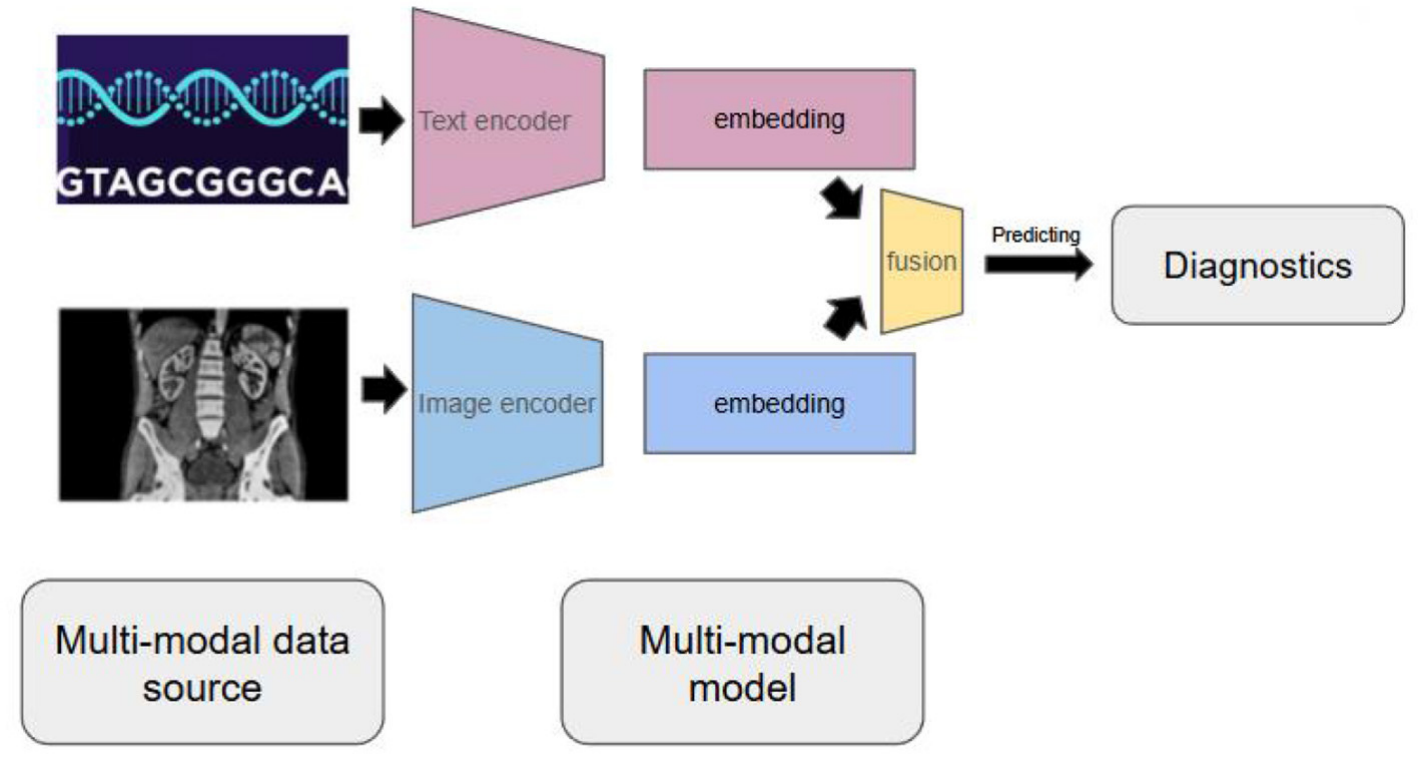
Multi-modal AI models can incorporate different data source and transform them into embedding and perform downstream tasks, such as predicting diagnostics.

Unlike traditional AI systems that focus on single data types, multi-modal AI can simultaneously process and integrate genomics sequences, medical imaging, electronic health records, and even social variants of health (see Figure 1). To be more specific, multi-modal AI models incorporate different types of data source and transform them into embedding by joining independent embedding of data sources (9). More recent models using data fusion layer to fuse multiple embedding into one embedding for downstream tasks (10), such as predicting diagnostics. This approach mirrors how physicians naturally think about patients—not as isolated genetic data, but as complex individuals whose health comes from multiple interacting factors.

Consider cancer treatment, where this convergence is already yielding remarkable results. Multi-modal AI systems can analyze tumor genomics alongside histopathological images, treatment history, and biomarkers to predict which patients will respond to specific immunotherapies (11, 12). Companies such as Tempus and Foundation Medicine are pioneering platforms that combine genomic profiling with clinical data analysis, enabling oncologists to make treatment decisions based on comprehensive molecular information. In cardiology, researchers have developed models that integrate genetics with risk variants of patients (13, 14). In neurology, researchers developed a multi-modal screening system for elderly neurological diseases (15).

Multi-modal approaches are particularly powerful for addressing health disparities. By incorporating social determinants of health alongside genomic data, these systems can better account for the environmental and socioeconomic factors that significantly influence health outcomes in different populations (16, 17). This is crucial for ensuring that precision medicine benefits all patients, not just those in affluent healthcare systems.

## Discussion

### Challenges

Despite the great potential, significant challenges remain. Data privacy and security concerns exist when integrating such comprehensive personal information. Patients' genomic and health data meed to be protected while still enabling the data sharing necessary for AI model development and validation. Governments aware the importance of user privacy in AI applications and proposed standards such as GDPR and HIPAA to protect users, and federated learning enable AI model training without disclosing original training data but still under the risk of privacy attack in parameter exchanges (18).

The interpretability of multi-modal AI models presents another critical challenge. As these systems become more sophisticated, understanding how they arrive at specific recommendations becomes important, but complex. Healthcare providers need AI tools that not only provide accurate predictions but can also offer clear explanations to patients and incorporated into clinical decision-making.

### Future directions

Looking ahead, the convergence of genomics and multi-modal AI will likely transform healthcare in ways we cannot imagine today. Chronic diseases could be predicted and prevented even before symptoms appear. Mental health conditions might be detected through subtle patterns in genomic data and behavioral indicators captured by wearable devices.

The integration of real-world evidence with genomic analysis will enable continuous refinement of treatment protocols, creating a learning healthcare system thatimproves outcomes for patients. Pharmacogenomics will become routine, reducing trial-and-error prescribing and reducing adverse drug reactions.

To advance precision medicine, we need sustained collaboration between healthcare providers, researchers, policymakers, and patients. Academic institutions need to train clinicians who can work effectively at the intersection of genomics, AI, and clinical care. Healthcare systems must develop the infrastructure necessary to capture, store, and analyze multi-modal health data securely.

Most importantly, we must keep patients at the center of this transformation. The ultimate measure of success will not be the accuracy of our algorithms or the value of our datasets, but whether we can deliver better health outcomes for individuals and communities.

The convergence of genomics and multi-modal AI represents more than a technological advancement—it embodies our evolving understanding of human health as a complex, dynamic system. By embracing this complexity rather than oversimplifying it, we can finally deliver on the promise of personalized medicine and even personalized healthcare. The revolution is already underway, and we need to ensure that the power of precision medicine serves humanity's highest aspirations for health and healing.
